# Phenolic-Based Discrimination between Non-Symptomatic and Symptomatic Leaves of *Aesculus hippocastanum* Infested by *Cameraria ohridella* and *Erysiphe flexuosa*

**DOI:** 10.3390/ijms241814071

**Published:** 2023-09-14

**Authors:** Agnieszka Hanaka, Sławomir Dresler, Wiesław Mułenko, Magdalena Wójciak, Ireneusz Sowa, Magdalena Sawic, Katarzyna Stanisławek, Maciej Strzemski

**Affiliations:** 1 Department of Plant Physiology and Biophysics, Institute of Biological Sciences, Faculty of Biology and Biotechnology, Maria Curie-Skłodowska University, Akademicka 19, 20-033 Lublin, Poland; 2 Department of Analytical Chemistry, Medical University of Lublin, Chodźki 4a, 20-093 Lublin, Poland; 3 Department of Botany, Mycology and Ecology, Institute of Biological Sciences, Maria Curie-Skłodowska University, Akademicka 19, 20-033 Lublin, Poland

**Keywords:** chestnut, pests, epicatechin, antioxidant capacity, UHPLC-MS, moth, fungus

## Abstract

The herbivore *Cameraria ohridella* (kingdom Animalia) and the pathogen *Erysiphe flexuosa* (kingdom Fungi) are considered pests and biotic stressors of *Aesculus hippocastanum* (chestnut trees). The impact of both pests on the accumulation of secondary metabolites in chestnut leaves was investigated. Specifically, the interactive effect of both pests on metabolite accumulation and their potential role in enhancing the resistance of chestnut trees to biological stress was the focus of this study. *Aesculus hippocastanum* leaves with varying degrees of *Cameraria ohridella* infestation and *Erysiphe flexuosa* infection were used in this research. Leaf samples were collected during the plant vegetative growth phase and evaluated for pest infection and secondary metabolite content. Eight main polyphenols were identified in the leaves: (1) neochlorogenic acid, (2) (−)-epicatechin, (3) procyanidin trimer A-type, (4) procyanidin tetramer A-type, (5) quercetin-3-*O*-arabinoside, (6) quercetin-3-*O*-rhamnoside, (7) kaempferol-3-*O*-arabinoside, and (8) kaempferol-3-*O*-rhamnoside. It was found that the accumulation of metabolites, primarily those derived from epicatechin and quercetin, during the initial vegetation phase (up to 11.05 or 09.05), strongly depended on the later degree of pest infection. The differences observed in the metabolite dynamics in the chestnut leaves, depending on the extent of infection, indicate the development of a metabolic response mechanism in chestnut trees to biological stress.

## 1. Introduction

*Aesculus hippocastanum* L., commonly known as horse chestnut, belongs to the Sapindaceae family and the Hippocastanoideae subfamily [1]. It can grow rapidly, reaching up to 36 m in height. Cultivated as an ornamental tree, it is often found in European cities, towns, parks, and woodlands [2] and is known as a medicinal, pharmaceutical, and cosmetic species [3]. This plant synthesizes metabolites representing different groups, e.g., secondary metabolites such as phenolic compounds, flavonoids, tannins, saponins, and coumarins [4,5,6,7,8,9] (Table 1). Overall, the highest concentration of chemicals is detected in its seeds, but they are also contained in the fruit, bark, leaves, buds, and embryonic callus tissue [6]. 

The chemical composition inside and outside the leaf and its physical parameters are crucial for plant susceptibility or resistance to infection. *Aesculus hippocastanum* leaves contain known chemicals with antifeedant properties against insects, e.g., polyphenolic compounds like (−)-epicatechin and procyanidins, whereas infection can be associated with a lower concentration of flavan-3-ols [2]. In addition to condensed tannins and their monomers (e.g., epicatechin) with their antifeedant function in *A. hippocastanum* leaves and bark, the other two antifeedants are esculin and fraxin, which are the most abundant in early spring and late autumn [9]. The epicuticular wax of mature leaves of *A. hippocastanum* consists mainly of triterpenols (53% of the wax) and lipids [10]. The triterpenols are represented by β-amyrin (20% of the wax), α-amyrin and lupeol (15% of the wax each), friedelan-3-ol, and friedelan-3-one (1.5% of the wax each). The main lipid classes are aldehydes (13%), fatty acids (8%), primary alcohols (7%), wax esters (7%), hydrocarbons (5%), and acetates (5%) [10]. It has been found that the wax composition on the abaxial and adaxial leaf sides may vary [10]. This variability may also partially participate in a plant’s reaction to infection. Also, physical parameters such as contact angle or the drop asymmetry coefficient (DA) are known to indirectly determine pathogen growth on leaves. The shape of a water drop on a leaf surface regulates the evaporation time [11]. As shown by the DA coefficient, the adaxial side of the *A. hippocastanum* leaf is wettable (0.014), whereas the abaxial side is highly wettable (0.016) [12]. A highly wettable surface allows for quicker dissipation of the water droplet, preventing some kinds of infection. On the other hand, climatic conditions also determine the level of potential infection, as a greater amount of water or a longer persistence of water on the leaf surface creates favorable conditions for plant pathogen growth [12].

*Aesculus hippocastanum* has two threatening pests: larvae of the moth *Cameraria ohridella* Deschka and Dimić, a leaf mining insect known as the horse chestnut leaf-miner, and mycelia of the fungus *Erysiphe flexuosa* (Peck) U. Braun and S. Takam [13,14,15]. However, in individual *A. hippocastanum* trees, the considerable variation in the susceptibility to infection has been observed to be independent of the age of the tree [14]. Morphological changes in the leaf tissues of horse chestnuts colonized by *C. ohridella* [16] and its developmental forms [17] have been described earlier. Depending on climatic conditions, up to four generations of this pest can appear [7]. The larvae mine the leaves between the epidermis layers [7]. At the beginning of larval feeding, characteristic orange spots in the leaf blade are observed; these epidermal cells have dark-red walls and orange cytoplasm [17]. This color may be an indicator of the presence of an increased level of tannins in cells [17]. However, *Erysiphe flexuosa* (previously classified as *Uncinula flexuosa*) frequently causes powdery mildew on *A. hippocastanum* trees [18]. Initially, small patches appear on leaves, and next, the infection involves both the upper and the lower leaf surfaces [18,19]. Leaf damage provoked by both invasive species *C. ohridella* and *E. flexuosa* influences photosynthetic productivity through the degradation of chloroplasts, a reduction in gas exchange, and limitation of the flowering and fruiting of horse chestnut trees [15,20]. The seeds of infected trees are characterized by lower germinability but a stable quantity [20,21]. Consequently, colonization by both pests causes premature leaf fall [7,18]. Among the defense-related compounds, tannins and polyphenols produced in plant tissues under stress are the most promising agents against pests [2,22]. It is advisable that these compounds should be further analyzed to extend our knowledge.

The goal of the study was to examine the influence of *Cameraria ohridella* pest infestation and *Erysiphe flexuosa* fungal infection on the content of the secondary metabolites (phenolic compounds) found in the leaves of *Aesculus hippocastanum*. The mechanism of attraction or resistance to pests based on the involvement of compounds produced by horse chestnut trees still requires elucidation. Verification of the chemical composition should help to discriminate between non-symptomatic (control) and symptomatic (with visual symptoms of leaf damage) leaves and to assess some of the chemicals as potential defensive substances.

## 2. Results and Discussion

### 2.1. Visualization of Damage to Horse Chestnut Leaves

Table 2 presents the dynamics of leaf damage. At the end of the experiment, except for the control, the highest level of *C. ohridella* infection was detected in all trees, whereas the highest *E. flexuosa* infection was noted in four (B–E) of the six trees. This means that individuals A and F were infected only/mainly by *C. ohridella,* while individuals B–E were characterized by both *C. ohridella* infestation and *E. flexuosa* infection. Visual symptoms of infection started to appear on the leaf surface from the fourth term of the collection period, i.e., at the end of May. Example photographs of the control and symptomatic leaves with whitish and brownish spots are presented in Supplementary Figure S1. When predicting the level of infection, its dynamics should be considered. For example, a rapid rise in leaf damage during the first three years of infection with a slight rise thereafter was determined in a previous study [23].

According to the literature, *A. hippocastanum* trees prefer warm and moist summers and warm winters [24] and demonstrate a weather–plant condition relationship. The occurrence of infection caused by *C. ohridella* and *E. flexuosa* increases the sensitivity of the trees to meteorological oscillations, resulting in premature defoliation [24,25]. Since similar weather conditions were recorded in Lublin (symptomatic trees) and Mircze (control trees), the study data suggest that weather is not exclusively responsible for the extent of leaf damage.

### 2.2. Identification and Quantification of Phenolics and the Level of Antioxidant Activity in Horse Chestnut Leaves

**Figure 1:**
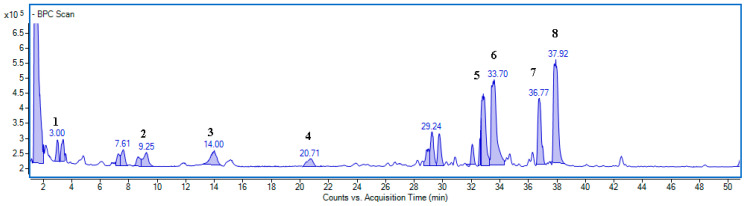
Representative base peak chromatograms (BPC) of the compounds in *A. hippocastanum* control leaves. (1) Neochlorogenic acid; (2) (−)-epicatechin; (3) procyanidin trimer A-type; (4) procyanidin tetramer A-type; (5) quercetin-3-*O*-arabinoside; (6) quercetin-3-*O*-rhamnoside; (7) kaempferol-3-*O*-arabinoside; (8) kaempferol-3-*O*-rhamnoside.

Resistance or susceptibility to pests is the result of plant–pest interactions. The chemical composition of leaves seems to be a prevailing agent in which communication takes place [26]. It is generally accepted that phenolic compounds representing secondary metabolites can modulate plant resistance against pests [22,27], and infection may induce accumulation of phenolics in the leaves of host plants [28]. Hence, their role and physiological activity is worth exploring. In the *A. hippocastanum* leaves, we detected eight specific phenolic compounds, which are presented in a chromatogram (Figure 1 and Figure S2) and characterized in Table 3.

The identity of a particular component was established based on a comparison of the retention time, UV spectrum, and MS ionization pattern with the standard. When no standard was available, the chemical formula of the component was deduced by comparing the difference between the mass data and theoretical values, ensuring a deviation below 5 ppm. The compound was identified based on the UV spectrum and mass data given in the literature [2,7]. The UV-Vis and MS spectra are presented in Figure S3 and Figure S4, respectively.

**Figure 2:**
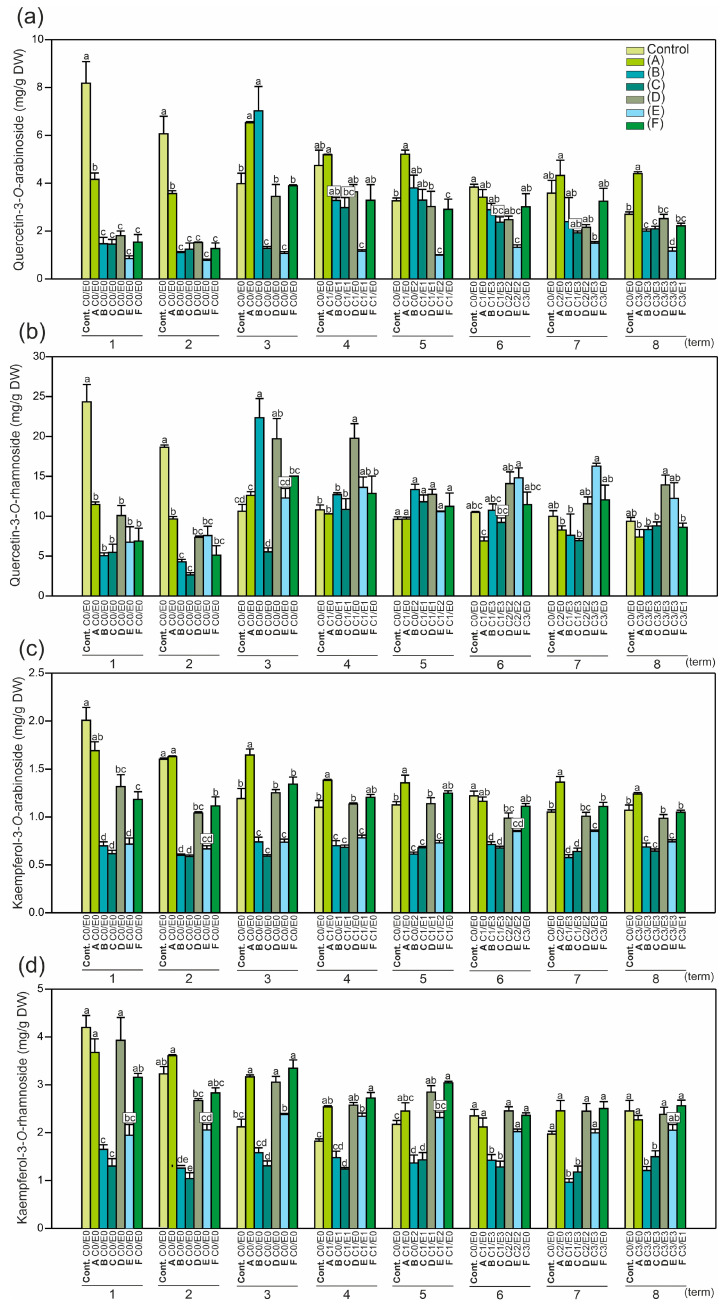
Mean content (±SE) (*n* = 3–4) of (**a**) quercetin-3-*O*-arabinoside, (**b**) quercetin-3-*O*-rhamnoside, (**c**) kaempferol-3-*O*-arabinoside, and (**d**) kaempferol-3-*O*-rhamnoside in the control and symptomatic leaves (A–F) of *A. hippocastanum* trees measured for eight collection periods (terms 1–8). Values followed by different letters within the same tree are significantly different (*p* < 0.05, Tukey’s test).

**Figure 3:**
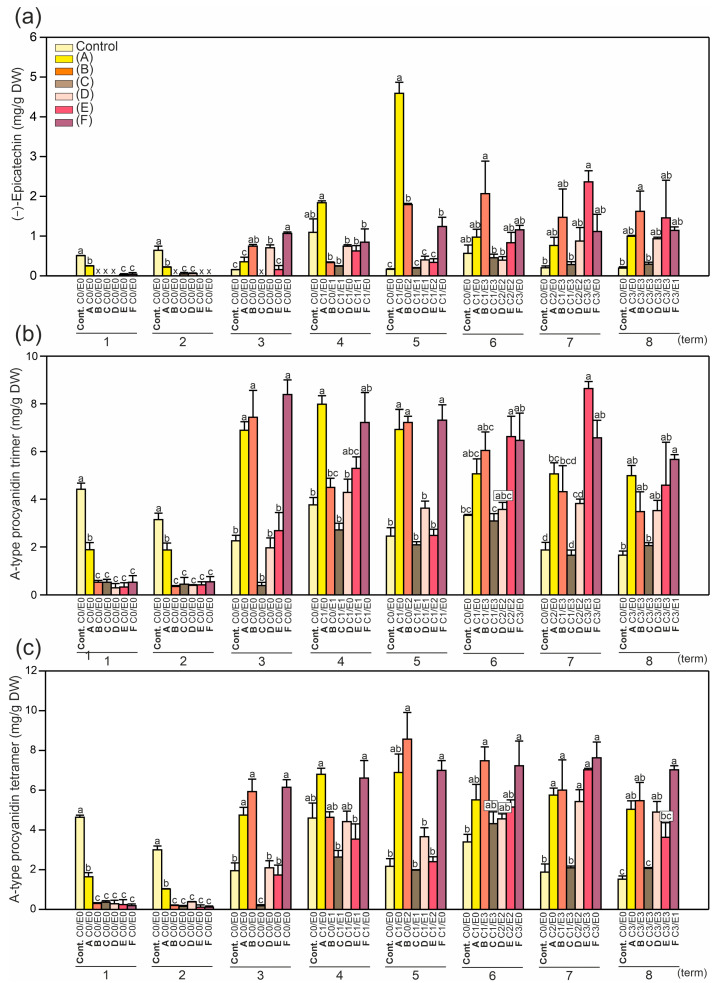
Mean content (± SE) (n = 3–4) of (**a**) (−)-epicatechin, (**b**) procyanidin trimer A-type, and (**c**) procyanidin tetramer A-type in the control and symptomatic leaves (A–F) of *A. hippocastanum* trees measured for eight collection periods (terms 1–8). Values followed by different letters within the same tree are significantly different (*p* < 0.05, Tukey’s test).

**Figure 4:**
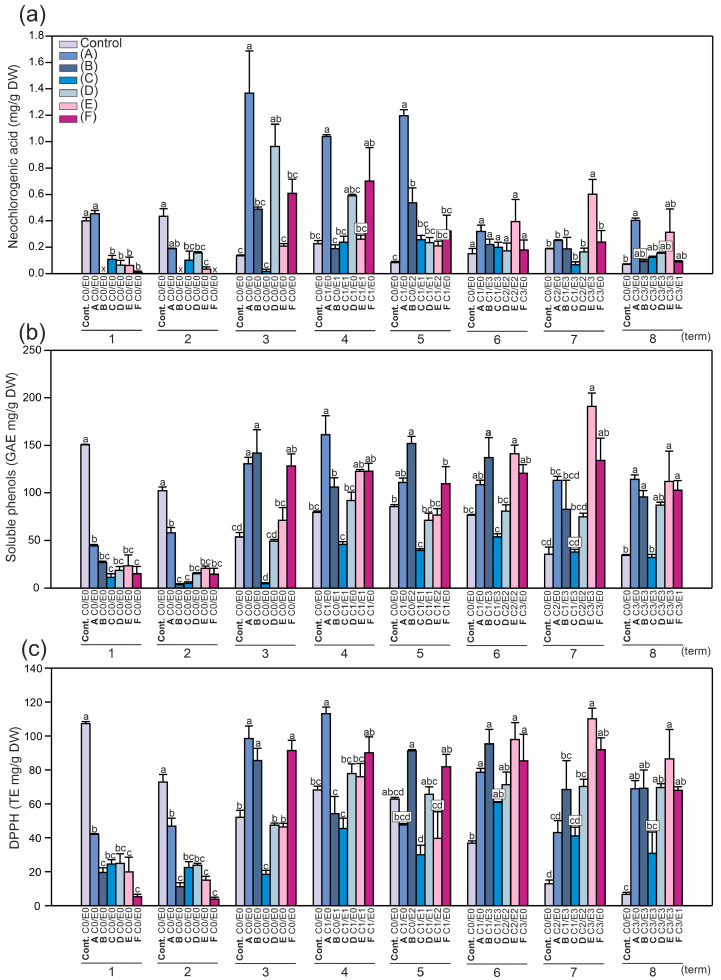
Mean content (± SE) (n = 3–4) of (**a**) neochlorogenic acid, (**b**) soluble phenols, and (**c**) DPPH levels in the control and symptomatic leaves (A–F) of *A. hippocastanum* trees measured for eight collection periods (terms 1–8). Values followed by different letters within the same tree are significantly different (*p* < 0.05, Tukey’s test).

**Figure 5:**
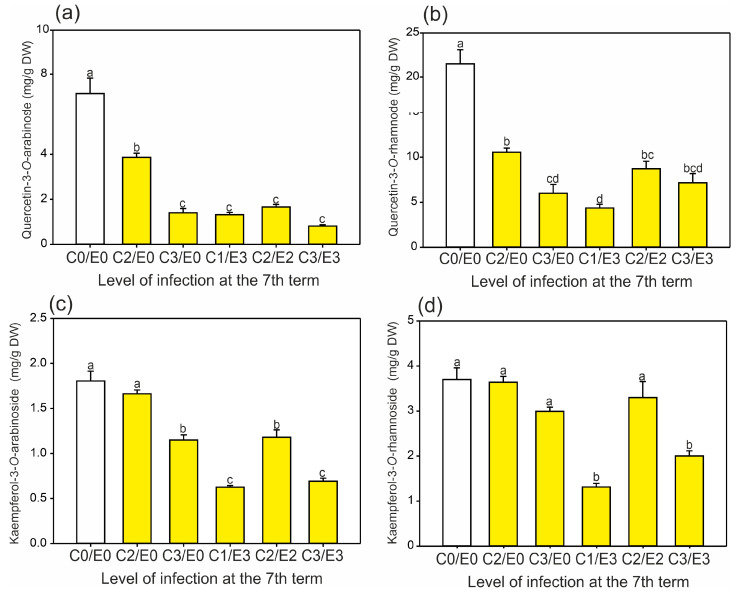
Relationship between the mean contents of (**a**) quercetin-3-*O*-arabinoside, (**b**) quercetin-3-*O*-rhamnoside, (**c**) kaempferol-3-*O*-arabinoside, and (**d**) kaempferol-3-*O*-rhamnoside in the *A. hippocastanum* leaves in the initial stage of vegetation (first and second terms) and further levels of infection intensity observed in the seventh term. Data are means ± SE (*n* = 6–8) from the first and second terms of leaf collection. Values followed by different letters are significantly different (*p* < 0.05, Tukey’s test).

**Figure 6:**
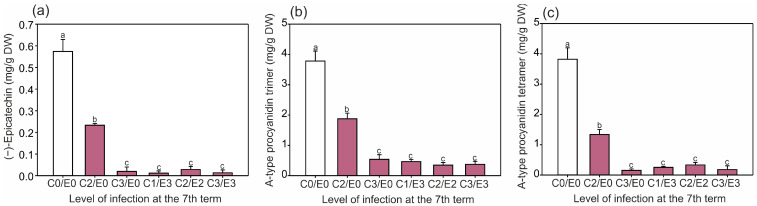
Relationship between the mean contents of (**a**) (−)-epicatechin, (**b**) procyanidin trimer A-type, and (**c**) procyanidin tetramer A-type in the *A. hippocastanum* leaves in the initial stage of vegetation (first and second terms) and the further level of infection intensity observed in the seventh term. Data are means ± SE (*n* = 6–8) from the first and second terms of leaf collection. Values followed by different letters are significantly different (*p* < 0.05, Tukey’s test).

**Figure 7:**
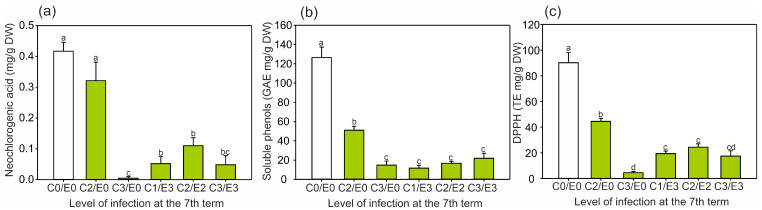
Relationship between the mean contents of (**a**) neochlorogenic acid, (**b**) soluble phenols, and (**c**) antioxidative capacity (DPPH) in the *A. hippocastanum* leaves in the initial stage of vegetation (first and second terms) and the further level of infection intensity observed in the seventh term. Data are means ± SE (*n* = 6–8) from the first and second terms of leaf collection. Values followed by different letters are significantly different (*p* < 0.05, Tukey’s test).

The collection period determined the content of secondary metabolites and the DPPH levels (Figure 2, Figure 3, Figure 4, Figure 5, Figure 6, Figure 7 and Figures S5–S7). The difference in the content of all the measured compounds between the first and last day (8th term) of the experiment was pronounced (Figure 2, Figure 3, Figure 4 and Figures S5–S7). Furthermore, the trends observed in the control leaves were opposite to those noted in the symptomatic leaves (A–F). In the control leaves, the reduction in the level of all the measured compounds between the first and the last day of the experiment was striking (from 18 to 58%). For example, a major decrease was found in the case of the kaempferol-3-*O*-arabinoside and rhamnoside forms: 5.3- and 5.8-fold, respectively; whereas the content of neochlorogenic acid was 1.8-fold lower (Figure 2 and Figure 4). In the symptomatic leaves, an increase in the content of these compounds was detected between the first and eighth terms. For example, the most pronounced increases were 8.5-fold for epicatechin, 13.9-fold for procyanidin trimer A-type, and 36.9-fold for procyanidin tetramer A-type (Figure 3). Also, epicatechin was the only examined compound that was not detected in half of the examined symptomatic leaves at the beginning of the vegetation period, which also makes it a good chemical marker for the prediction of plant conditions in the near future.

Moreover, in all the tested leaves (control and symptomatic), the level of compounds in the first collection period was very similar to that in the second collection period (Figure 2, Figure 3, Figure 4, Figure 5, Figure 6, Figure 7 and Figures S5–S7). In the third collection period, substantial changes in the content of the compounds between the control and symptomatic leaves were shown, with an increase in the symptomatic leaves compared to the control (Figure 2, Figure 3, Figure 4 and Figures S5–S7). In all the tested leaves, the content of the compounds in the seventh and eighth terms was similar and showed reduced dynamics of modifications during the last 50 days of the experiment. The conclusion is that the higher the content of the measured compounds in the first two terms, the lower the possibility of infection during the entire vegetation period (Figure 2, Figure 3, Figure 4 and Figures S5–S7). In a study conducted by Materska et al. [27], a higher content of total phenolic compounds and a higher antiradical activity were detected in infected leaves, whereas this tendency in our research was noted only after the third collection period (opposite to the first two collection periods) (Figure 2, Figure 3, Figure 4 and Figures S5–S7). Therefore, our results are in contradiction with the high level of total phenolics determined at the beginning of May in the susceptible leaves compared to the resistant ones [29].

Additionally, it was noted [27] that the plant condition, especially in the initial growing season, strongly depended on habitat factors such as sunlight exposure, water supply, or soil conditions. The distribution and concentration pattern of the chemicals synthesized in the leaves was linked also to the availability of nutrients, the place of a leaf on the tree, or the impact of environmental pollutants [21]. The last factor may be the crucial cause of the differentiation between the control plants from the rural area and the symptomatic plants from the city analyzed in this research.

In the literature, phenolic compounds have been examined in different parts of *A. hippocastanum*, e.g., the leaves [2,27,29], flowers [30], fruit pulp, skin, husk [4], or seed kernels [31]. The extracts may be useful not only for plant protection against insect invasions in urban and rural environments but also in various industries such as food production, cosmetics, and pharmaceuticals. Our results (Figure 2, Figure 3, Figure 4 and Figures S5–S7) confirm that the clear changes in the leaf phytochemistry detected in the middle of May (from the third collection period) preceded the visual leaf damage (from the fourth collection period) (Table 2).

The content of both quercetin derivatives (Figure 2a,b), the epicatechin and procyanidin forms (Figure 3a–c), was high in the two first collection periods (first and second) only in the control leaves, which is in line with the results reported by Oszmiański et al. [2]. Moreover, in our experiment, the quercetin-3-*O*-rhamnoside content increased to 24 mg/g DW in the studied samples, whereas in other reports, it did not exceed 7 mg/g DW [27]. Our results showed a significantly higher difference in the epicatechin content between the control (0.56 mg/g DW) and the symptomatic leaves (up to 2.07 mg/g DW), reaching an up to 3.7-fold increase in late June (sixth term) (Figure 3b). However, in the literature, its level was 1.2-fold higher in control leaves (9.5 mg/g DW) than in the infected ones (7.77 mg/g DW) examined in June [2].

The same correlations were found for the content of soluble phenols and the DPPH levels (Figure 4b,c), with the highest amounts detected in the control samples at the beginning of the vegetation period. During the experiment, the content of soluble phenols in the control leaves was reduced 4.4-fold (from 151 to 34 mg GAE/g DW), and the DPPH levels were 13.7-fold lower (from 107 to 7.8 mg TE /g DW). From the third collection period, the content of the compounds measured in the control leaves was similar until the end of the experiment (Figure 2 and Figure 3), while the DPPH levels were steadily reduced (Figure 4b). In the symptomatic leaves, the most pronounced difference was noted in the content of soluble phenols (up to 6.8-fold: from 15.1 to 102.8 mg GAE/g DW; tree F) and the DPPH levels (12.8-fold: from 5.3 to 68 mg TE/g DW) between the first and eighth terms of the experiment (Figure 4b,c). Regardless of the infection, the level of total phenolics was generally similar to the data presented in another report, which indicated a range above 40 and did not exceed 100 mg GAE/g DW [27].

It was noted that quercetin derivatives (Figure 5a,b) were accumulated in the non-symptomatic leaves in both the first and second terms during further vegetation (seventh term). Interestingly, a similar tendency was also observed for the kaempferol derivatives (Figure 5c,d); however, the leaves that were heavily infected by *E. flexuosa* (E3) in the mature stage were characterized by significantly lower accumulation of these phenolics in the initial growth stage compared to the other trees (Figure 5d).

The content of both quercetin derivatives in the leaves was statistically reduced at the beginning of the growing season in the leaves infected later (Figure 5a,b). Both the kaempferol derivatives were less responsive to the later presence of the pests (Figure 5c,d) than the quercetin derivatives (Figure 5a,b). In the group of the kaempferol derivatives, the arabinoside content was reduced in almost all the samples (except C2E0), while the rhamnoside content was lower only in two types of samples, C1E3 and C3E3. The content of both rhamnoside forms (Figure 5a,d) was higher than that of both arabinoside forms (Figure 5a,c), which is in accordance with other data [2]. Oszmiański et al. [2] found that the content of quercetin-3-*O*-rhamnoside compared to quercetin-3-*O*-arabinoside was 3.3- and 3.2-fold higher in the control and infected leaves, respectively. These findings are similar to our results, which showed 3-fold higher values in the control and in the range of 2.7–8.8 (mean 4.9) in the leaves infected later. A similar tendency, but with lower values, was proved for the content of kaempferol-3-*O*-rhamnoside compared to kaempferol-3-*O*-arabinoside. Oszmiański et al. [2] reported 4- and 3.4-fold differences in the content of the former derivative between the control and infected leaves, respectively, whereas our research showed 2-fold higher content in the control and a range of 2.1–2.9 (mean 2.5) in the leaves infected later.

The epicatechin and procyanidin contents in the leaves (Figure 6a–c) exhibited the same tendency as the quercetin-3-*O*-arabinoside content (Figure 5a). Therefore, despite the later infection, the content of these compounds was lower than in the non-symptomatic samples. It is worth mentioning that the content of the compounds in the C2E0 samples was higher than in any other infected leaf samples (Figure 5, Figure 6 and Figure 7). Our results showed a 2.5 to 48-fold higher content of epicatechin in the symptomatic leaves compared to the non-symptomatic ones (Figure 6a). There is evidence that the toxicity of catechins to pests may be strongly connected to the production of high levels of reactive oxygen species [32]. Furthermore, tannins may inhibit the activity of enzymes that play a key role in insect feeding [17].

The neochlorogenic acid content in the leaves (Figure 7a) was similar to the amount of kaempferol-3-*O*-arabinoside (Figure 5c). Most of the leaves infected later (except C2E0) had significantly lower content of neochlorogenic acid than non-symptomatic leaves, with the greatest reduction detected in the C3E0 samples (Figure 7a).

Moreover, the levels of all the compounds analyzed in this study were directly correlated to the DPPH levels (Figure 4c), e.g., the higher the content of secondary metabolites, the higher the content of DPPH. As a consequence of the highest synthesis of phenolics and tannins, elevated production of reactive oxygen species was observed, which is in accordance with the literature [32]. In the case of epicatechin, the relationship between pest infection in the further growth stage and the content of these metabolites in the initial stage was much more significant (Figure 6a). Except for the C2E0 leaf samples (approx. 2.5 lower content of epicatechin than in the non-symptomatic leaves), the C3E0 samples and leaves infected by both pests in the later growth stage contained significantly lower amounts of epicatechin derivatives, i.e., approx. up to 30-fold lower than in the non-symptomatic samples. A similar pattern was observed in the case of neochlorogenic acid, total polyphenolics, and antioxidant properties (Figure 7).

The content of soluble phenols (Figure 7b) had the same tendency as that demonstrated for epicatechin and procyanidin (Figure 6a–c). Significantly lower contents were found for all the leaves infected later compared to the non-symptomatic samples (Figure 7b). Similarly, the DPPH levels in the leaves (Figure 7c) were lower in all the samples of leaves infected later compared to the non-symptomatic samples, with the highest content in C2E0 and the lowest content in the C3E0 samples. Interestingly, the most intense infection with *C. ohridella*, despite the presence of *E. flexuosa*, caused the strongest reduction in the neochlorogenic acid content and DPPH levels (statistically indifferent values between the C3E0 and C3E3 leaves) (Figure 7a,c). Among the infected leaves, the highest content of the measured compounds was detected in the C2E0 samples (Figure 5, Figure 6 and Figure 7).

Even though significant changes in the level of almost all compounds were detected in the third term/period, the first visible symptoms of infection appeared in the fourth term. Immediately before the appearance of visible symptoms of infection, the plants begin to synthesize higher contents of compounds that may provide chemical protection. Moreover, the healthy condition of the *A. hippocastanum* leaves, manifested by a high content of secondary metabolites in the early growing season (first and second), resulted in a limitation of infections, which has also been suggested in other reports [27]. The chemicals chosen in our experiment were relevant indicators of the forthcoming infections. Contrary to our results, which showed a lower level of the total phenolic content at the beginning of the growing season but a higher level in the infected leaves from the third to the last day of the experiment, other scientists have proven that the total phenolic content in the relatively susceptible *A. hippocastanum* trees was similar or higher than in the relatively resistant *A. hippocastanum* [29]. This may suggest that rather than the overall pool of phenolics, a specific phenolic composition is responsible for the different susceptibility of the trees.

Various tendencies between the content of phenolic compounds and tannins after infection have been presented in the literature. Some studies have described these compounds as powerful defensive agents with a significant effect on the inhibition of the negative activity of pests [29,33,34], which is in line with our results. On the other hand, the lack of differences in the chemical composition between non-symptomatic and infected leaves has also been reported [27]. This may be explained as the impact of larval foraging that did not allow for accumulation of the studied compounds in the leaves, despite the direct inhibition of foraging induced by these compounds [27].

The differences in the chemical composition of the leaves allow for the identification of substances that may be important for the interaction between pests and trees. The present results suggest that the first stage of the defense mechanism involved rapid synthesis of phenolic compounds with elevated levels of DPPH (from the third collection period). The effort to reduce infection was ineffective, as demonstrated in our studies and the literature [35]. We assumed that the non-symptomatic horse chestnut trees exhibited higher contents of phenolic compounds that may have a harmful effect on the pest population and do not contain nutritional compounds for the pests [7]. Elevated contents of phenolics, especially epicatechin and procyanidins in leaves, is suggested to explain their greater resistance to *C. ohridella* [2]. This statement is in agreement with our results, but only when considering their levels at the beginning of the vegetation period.

Similar to other studies [29], we demonstrated an increase in the phenolic content in the *A. hippocastanum* leaves with the development of the pests, which may be considered to be a manifestation of chemical defenses against infection. Finally, the elevated synthesis observed in our research was ineffective in plant protection, which is in agreement with the literature data [29]. This might result from the fact that pests can have an effective defense system, which includes a broad set of enzymes (e.g., glutathione-S-transferase) responsible for detoxification of the chemicals produced by the host plants [21]. In contrast to the above-described insufficient plant protection strategy connected with the production of chemicals, some results have revealed its effectiveness [27]. However, the data presented by other researchers [2] also showed a decrease in the chemical content as a consequence of pest activity, which does not support our statement. It seems that monitoring of even only one compound, for example, the level of epicatechin in late April or early May, may provide a reliable indicator/predictor of the plant condition in the near future. Such results have proved that leaf phytochemistry is the best way to evaluate and predict plant health status before some changes are visible to the human eye. Furthermore, other groups of metabolites should also be analyzed in plant response to unfavorable conditions, such as saponins with their cytotoxic activity [5].

Protection of plants against the effects of diseases requires complex management. Guidelines should be based on good practices of plant monitoring and application of relevant recommendations for a comprehensive and sustainable strategy [36]. Effective management recommendations include regular monitoring, which facilitates rapid detection and identification of diseases, the use of innovative tools for mapping and creating digital databases of pathogens, systematic removal of fallen infected parts of plants, and burning removed parts of infected plants [36].

### 2.3. Principal Component Analysis (PCA)

**Figure 8:**
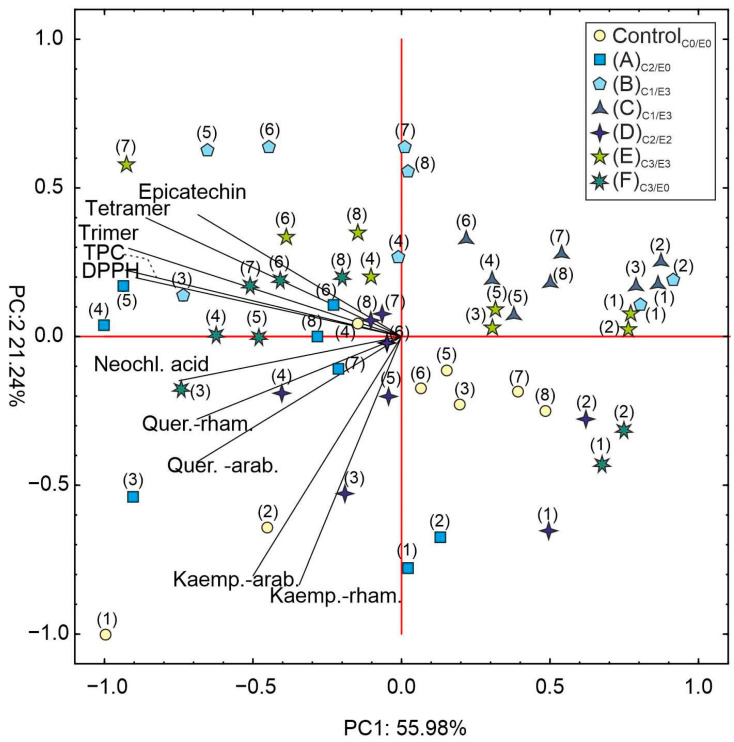
Principal component analysis (PCA) of the mean content of the analyzed compounds in the leaves of *A. hippocastanum* trees (control and symptomatic A–F) measured at eight collection periods (1–8) compared with the infection intensity evaluated at the seventh collection period (7th term). The analyzed compounds: quercetin-3-*O*-arabinoside (Quer.-arab.), quercetin-3-*O*-rhamnoside (Quer.-rham.), kaempferol-3-*O*-arabinoside (Kaemp.-arab.), kaempferol-3-*O*-rhamnoside (Kaemp.-rham.), (−)-epicatechin (Epicatechin), procyanidin trimer A-type (Trimer), procyanidin tetramer A-type (Tetramer), neochlorogenic acid (Neochl. acid), soluble phenols (TPC), and antioxidative capacity (DPPH).

Regarding the PCA analysis of the *A. hippocastanum* leaf samples, as shown in Figure 8, the examination aimed to unveil potential relationships among the assessed variables. The first two principal components, PC1 and PC2, collectively accounted for 77.22% of the total variance. These values were higher than those reported in a study conducted by Oszmiański et al. (2014) [2], where PC1 and PC2 together explained 30.69% of the total variability.

Although the unequivocal separation between the groups was somewhat limited in our research, an inclination towards separating the individuals within the two-dimensional space was demonstrated. Intriguingly, specific patterns emerged in correlation with the collection periods. The non-symptomatic leaves assessed during the initial two collection periods exhibited results that negatively correlated with PC1 and PC2. Conversely, the results from the subsequent collection periods (3–8) displayed a positive correlation with PC1 and a negative correlation with PC2. In terms of the first and second collection periods, a negative correlation was observed for the non-symptomatic leaves, while all the infected leaves displayed a positive correlation with PC1. Furthermore, among the six individuals (A–F), three (A, D, and F) displayed a negative correlation with PC2, whereas the other three (B, C, and E) exhibited a positive correlation. Regarding the third to eighth collection periods, the leaf samples generally demonstrated a positive correlation with PC2, except for 3F and 3–6D. Additionally, the compounds displayed a negative correlation with PC1 and exhibited both negative and positive correlations with PC2 (Figure 8).

## 3. Materials and Methods

### 3.1. Plant Material and Sample Collection

We selected the horse chestnut localities based on observations made in the previous year. Hence, the experiment was performed in the field in two different rural and urban localities in Poland, i.e., Mircze village (Hrubieszów district, Lublin province; 23°54′ E 50°39′ N) and Lublin city (51°14′35.8″ N 22°32′29.0″ E, 51°16′17.2″ N 22°33′00.1″ E and 51°14′37.4″ N 22°32′27.7″ E). We examined seven trees: six independent trees infested by *C. ohridella* larvae and/or *E. flexuosa* fungus (symptomatic, labelled A–F) and one control tree (non-symptomatic). Three leaves were collected from each tree in each sampling period. The leaves were collected at eight time points during 100 days of the vegetation period, from 29.04 or 01.05 (in Lublin and Mircze, respectively) to 07.08 or 09.08 (in Lublin and Mircze, respectively), as shown in Table 4. Finally, the leaves were assigned to two groups according to the presence of the pest/s: (I) non-symptomatic from Mircze area and (II) symptomatic—naturally infected with *C. ohridella* or with both *C. ohridella* and *E. flexuosa* from the Lublin area. After collection, the leaves were air dried for the chemical analyses. Data on the weather conditions in Lublin in the year of plant material collection are presented in Table S1 [37].

### 3.2. Scoring Damage to Horse Chestnut Leaves

The infection with the pests was recognized based on microscopic analyses using both light (Olympus BX 53, Tokyo, Japan) and scanning electron (SEM; TESCAN, VEGA3 LMU, Brno, Czech Republic) microscopes. The condition of the infected leaves was evaluated based on their appearance. The infected leaf area was calculated on a four-point scale as in Sucharzewska et al. [13] and Pocock and Evans [23] using ImageJ (ImageJ developers, version 1.52n. Wayne Rasband, National Institutes of Health, Bethesda, MD, USA).

The extent of leaf damage caused by *Cameraria ohridella* or infestation with *Erysiphe flexuosa* was evaluated as follows: 0—no damage, no evidence of moth attack or infestation, the leaf is completely green; 1—weak damage, up to 20% of the leaf surface damaged, some whitish/brown patches on the leaf; 2—medium damage, in the range 21–40%, there is more green than damaged leaf; 3—severe damage, above 40%, it is impossible to decide whether the leaf is green or damaged, whitish/brown patches may cover more than half of the leaf.

### 3.3. Determination of the Specific Polyphenol Content

The content of phenolic compounds was determined for methanolic extracts prepared using the following procedure. Two hundred milligrams of dry leaves (only parts without visible damage symptoms) were powdered using a laboratory grinder IKA A11 (IKA-Werke, Staufen, Germany) and transferred into 10 mL tubes. Subsequently, the sample was subjected to extraction three times, each with a fresh portion of 80% methanol (3 mL, 2 mL, and 2 mL). Each time after the application of the fresh methanolic solution, ultrasonification was carried out using an ultrasonic bath operating at a frequency of 35 kHz (Sonorex RK 512 H, Bandelin, Berlin, Germany) at room temperature for 10 min. The resulting extracts (after three extractions of the same sample) were combined, centrifuged using a Sigma 1-16K laboratory microcentrifuge (Sigma Laborzentrifugen GmbH, Osterode am Harz, Germany) at 10,000× *g* for 5 min, and then adjusted to a final volume of 5 mL in volumetric flasks. Prior to chromatographic analysis, the extracts were filtered through a 0.22 µm membrane filter.

An ultra-high performance liquid chromatograph (UHPLC) Infinity Series II with a DAD detector and an Agilent 6224 ESI/TOF mass detector with electrospray ionization (ESI) (Agilent Technologies, Santa Clara, CA, USA) was used to analyze the phenolic compounds in the leaf extracts [38]. The separation was conducted using an RP18 reversed-phase column Titan (Supelco, Sigma-Aldrich, Burlington, MA, USA) (10 cm × 2.1 mm i.d., 1.9 µm particle size). Acetonitrile with 0.05% of formic acid (solvent A) and water with 0.05% of formic acid (solvent B) at a flow rate of 0.2 mL/min were used as the mobile phase according the following gradient program: 0–30 min from 90% to 80% A (from 10 to 20% B) and 30–50 min from 80% to 75% A (from 20% to 25% B).

MS-grade formic acid and MS-grade acetonitrile were purchased from Sigma-Aldrich (St. Louis, MO, USA). The water was deionized and purified using Ultrapure Millipore Direct-Q^®^ 3UV-R (Merck KGaA, Darmstadt, Germany).

The thermostat temperature was 30 °C. DAD chromatograms were recorded from 200 to 400 nm. The ESI parameters were as follows: drying gas temperature 325 °C, drying gas flow 8 L min^−1^, nebulizer pressure 30 psi, capillary voltage 3500 V, skimmer voltage 65 V, and fragmentator voltage 200 V. Ions were acquired from 100 to 1200 m/z in the negative mode. Quantification was carried out based on the external calibration method using calibration curves obtained for epicatechin, neochlorogenic acid, and the corresponding flavonoid aglycones (quercetin and kaempferol) standards (Sigma-Aldrich). Glycosides were determined based on the curves of the corresponding aglycones. Procyanidins were calculated based on the calibration curve for epicatechin. The curves were prepared by injecting 5 µL of solutions with different concentrations of the above-mentioned analytes. The formulas of the curves are included in the Supplementary Materials (Table S2).

### 3.4. Determination of the Total Phenolic Content and Antioxidant Capacity

The total phenolic content (TPC; soluble phenols) was measured spectrophotometrically [39,40]. In this test, in addition to the Na_2_CO_3_ solution (100 mg/L), Folin–Ciocalteau reagent (Sigma-Aldrich, Poznań, Poland) was used, and the TPC content was measured after dark incubation at 750 nm and expressed as a gallic acid (Sigma-Aldrich, Poznań, Poland) equivalent (GAE).

The total antioxidant capacity was measured spectrophotometrically, applying the 2,2-diphenyl-1-picryl-hydrazyl-hydrate (DPPH) (Sigma-Aldrich, Poznań, Poland) assay, and expressed as a Trolox (Sigma-Aldrich, Poznań, Poland) equivalent (TE) [39,40].

### 3.5. Statistical Analysis

During the experiment, three-four randomly chosen leaves from one tree per collection period were analyzed. The statistical analyses were performed using the Statistica ver. 13.3.0 3 software (Tibco Software Inc., Palo Alto, CA, USA). The differences between the average accumulation of the metabolites observed in the first and second terms in relation to the further infection level (seventh term) were estimated using a one-way ANOVA. Also, a one-way ANOVA was applied to evaluate the impact of the collection terms within the same localization. The statistical differences between the groups were estimated using Tukey’s post-hoc test. The differences were considered statistically significant when the *p*-values were less than 0.05. 

Additionally, a PCA was conducted to confirm the relationships among the analyzed variables. The PCA was constructed based on the individuals representing mean values from three-four independent measurements of the same individual. A visualization of the PCA analysis results was conducted using the Biplot Statistica Visual Basic extension (StatSoft, Kraków, Poland) operating within the Statistica ver. 13.3.0 3 software environment (Tibco Software Inc., Palo Alto, CA, USA). The heat maps were constructed based on standardized data using GraphPad Prism version 8.0.0 (GraphPad Software, San Diego, CA, USA).

## 4. Conclusions

Phenolic compounds are secondary metabolites involved in the defense mechanisms of plants against such herbivorous (insect larvae) and pathogenic (fungus mycelia) pests as *C. ohridella* and *E. flexuosa*, respectively. We focused on comparing the phenolic content in leaves collected from symptomatic (*C. ohridella-* and *E. flexuosa*-infected) and non-symptomatic *A. hippocastanum* plants. We proved that the contents differed considerably between the non-symptomatic and symptomatic leaves of *A. hippocastanum* in a time-dependent manner. We found a significantly higher content of phenolic compounds in the non-symptomatic *A. hippocastanum* leaves at the beginning of the growing season, with significantly lower synthesis from the third collection period compared to the symptomatic leaves. This strategy protected the plants from infection. The opposite trend was found in the symptomatic leaves. We observed an elevation in the content of these chemicals in the third collection period, just before visible symptoms of infection appeared in the leaves. It seems that the maintenance of the synthesis of the analyzed compounds at a high level did not protect the leaves from the final limitation of the damaged area. Therefore, the efficient mechanisms of plant protection against infection must be activated at an appropriate time. As suggested by our studies, the initially high phenolics level (at the beginning of the vegetation period) is crucial for pest-free leaves.

Verification of the chemical composition should help to discriminate between non-symptomatic and symptomatic leaves and assess some chemicals as potential defensive substances. Based on our study, to estimate the probability of horse chestnut leaves being infected, one well-selected chemical representative can be chosen instead of measuring the whole set of compounds. We propose, for example, epicatechin as a reliable early biomarker (predictor) of leaf susceptibility to later infection during the vegetation period. In the near future, it may be possible to take action against infection in advance before its signs appear on leaves. This chemical monitoring approach will provide an opportunity to prevent or reduce the extent of infection more effectively.

Nevertheless, additional investigations are needed to help identify deeper correlations between the pest attack and the content of phenols throughout the entire horse chestnut vegetation season. The present results can provide a relevant background for further phytochemical studies on a larger population of *A. hippocastanum*.

## Figures and Tables

**Table 1:** Groups of metabolites and compounds detected in *Aesculus hippocastanum* [4,5,6,7,8,9].

Groups of Metabolites	Examples of Compounds
Fatty acids	Lauric, myristic, palmitic, stearic, arachic, and oleic acids
Organic acids	Oxalic, quinic, malic, citric, ascorbic, and shikimic acids
Carotenoids	Aesculaxanthin, lutein, and citraurin
Polyprenols	Undecaprenol, tridecaprenol, dodecaprenol, and castoprenol
Triterpene glycosides or saponins	Aescigenin, hippocaesculin and barringtogenol, the mix collectively called aesculin, aescin or escin
Coumarin derivatives	Esculetin; coumarin glucosides: esculin and fraxin
Flavonoids	Glycosides of quercetin, leucocyanidin, procyanidin, and kaempferol

**Table 2:** Dynamics of horse chestnut visual leaf damage caused by *C. ohridella* and *E. flexuosa* (0–3). Leaves of the control and symptomatic (A–F) trees were collected during eight collection periods (terms 1–8): 1—01.05 or 29.04; 2—11.05 or 09.05 (lasted 10 days); 3—21.05 or 19.05 (lasted 10 days); 4—31.05 or 29.05 (lasted 10 days); 5—10.06 or 08.06 (lasted 10 days); 6—20.06 or 18.06 (lasted 10 days); 7—15.07 or 13.07 (lasted 25 days); and 8—09.08 or 07.08 (lasted 25 days). The same number indicates the same level of infection: 0—no damage; 1—weak; 2—medium; and 3—severe damage.

Infection		*C. ohridella*						*E. flexuosa*					
	Term	1–3	4	5	6	7	8	1–3	4	5	6	7	8
Individuals													
Control		0	0	0	0	0	0	0	0	0	0	0	0
A		0	1	1	1	2	3	0	0	0	0	0	0
B		0	0	0	1	1	3	0	1	2	3	3	3
C		0	1	1	1	1	3	0	1	1	3	3	3
D		0	1	1	2	2	3	0	0	1	2	2	3
E		0	1	1	2	3	3	0	1	2	2	3	3
F		0	1	1	3	3	3	0	0	0	0	0	1

**Table 3:** Specialized metabolites identified in the *A. hippocastanum* leaf extracts using UHPLC-ESI/TOF.

Peak No	Rt (min)	Observed Ion Mass [M-H]-/(Fragments)	Δ ppm	Formula	Identified Metabolite
1	3.01	353.08798 (191,179)	0.49	C_16_H_18_O_9_	Neochlorogenic acid *
2	9.25	289.07189	0.44	C_15_H_14_O_6_	(−)-Epicatechin *
3	14.01	863.18319 (289)	0.35	C_45_H_36_O_18_	Procyanidin trimer A-type
4	20.71	1153.26211 (289)	0.16	C_60_H_50_O_24_	Procyanidin tetramer A-type
5	32.84	433.07788 (300)	0.56	C_20_H_18_O_11_	Quercetin-3-*O*-arabinoside
6	33.70	447.09387 (300)	1.31	C_21_H_20_O_11_	Quercetin-3-*O*-rhamnoside
7	36.77	417.08293 (285)	0.50	C_20_H_18_O_10_	Kaempferol-3-*O*-arabinoside
8	37.92	431.09871 (285)	0.79	C_21_H_20_O_10_	Kaempferol-3-*O*-rhamnoside

* The identification was confirmed using the standard, while the other components were identified based on the literature data [2,7].

**Table 4:** Dates of collection of non-symptomatic (Mircze) and symptomatic (Lublin) leaves in Poland.

Collection Period	Time Span between Collection Periods [Days]	Date of Collection of Non-Symptomatic Leaves in Mircze	Date of Collection of Symptomatic Leaves in Lublin
1	-	01.05	29.04
2	10	11.05	09.05
3	10	21.05	19.05
4	10	31.05	29.05
5	10	10.06	08.06
6	10	20.06	18.06
7	25	15.07	13.07
8	25	09.08	07.08

## Data Availability

The data are available upon request.

## Supplementary Materials

The following supporting information can be downloaded at: https://www.mdpi.com/article/10.3390/ijms241814071/s1.
