# Supplementary material for: Phenolic-Based Discrimination between Non-Symptomatic and Symptomatic Leaves of Aesculus hippocastanum Infested by Cameraria ohridella and Erysiphe flexuosa
Source: Int J Mol Sci. 2023 Sep 14;24(18):14071. doi: 10.3390/ijms241814071 (PMC10531086; doi:10.3390/ijms241814071)
Supplement: Supplementary file 1 [file ijms-24-14071-s001.zip › ijms-2562336-SI.pdf]

SUPPLEMENTARY MATERIALS

Phenolic-based discrimination between healthy and *Cameraria ohridella*- and *Erysiphe flexuosa*-infected leaves of *Aesculus hippocastanum*

Agnieszka Hanaka [1*], Sławomir Dresler [1,2*], Wiesław Mułenko [3], Magdalena Wójciak [2], Ireneusz Sowa [2], Magdalena Sawic [1], Katarzyna Stanisławek [1], Maciej Strzemski [2]

[1] Department of Plant Physiology and Biophysics, Institute of Biological Sciences, Maria Curie-Skłodowska University, Akademicka 19, 20-033 Lublin, Poland

[2] Department of Analytical Chemistry, Medical University of Lublin, Chodźki 4a, 20-093 Lublin, Poland

[3] Department of Botany, Mycology and Ecology, Institute of Biological Sciences, Maria Curie-Skłodowska University, Akademicka 19, 20-033 Lublin, Poland

* Correspondence: agnieszka.hanaka@mail.umcs.pl (A.H.); slawomir.dresler@umlub.pl (S.D.)

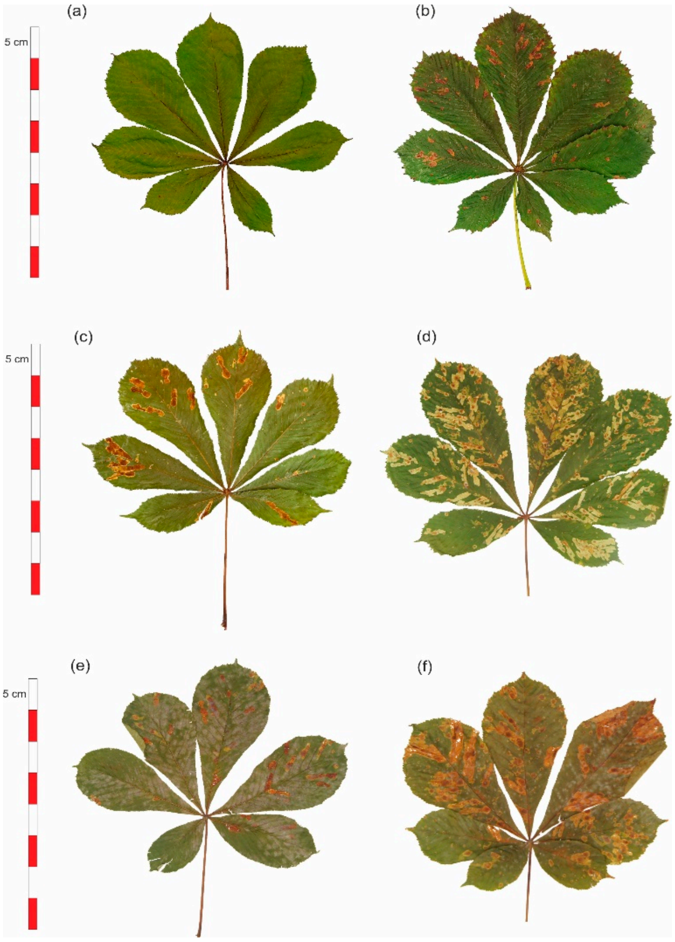

Figure S1. Representative photos of *Aesculus hippocastanum* leaves collected at the 7th collection period. Level of infection: (a) control, (b) and (c) C1E0, (d) C3E0, (e) C1E3, (f) C2E2.

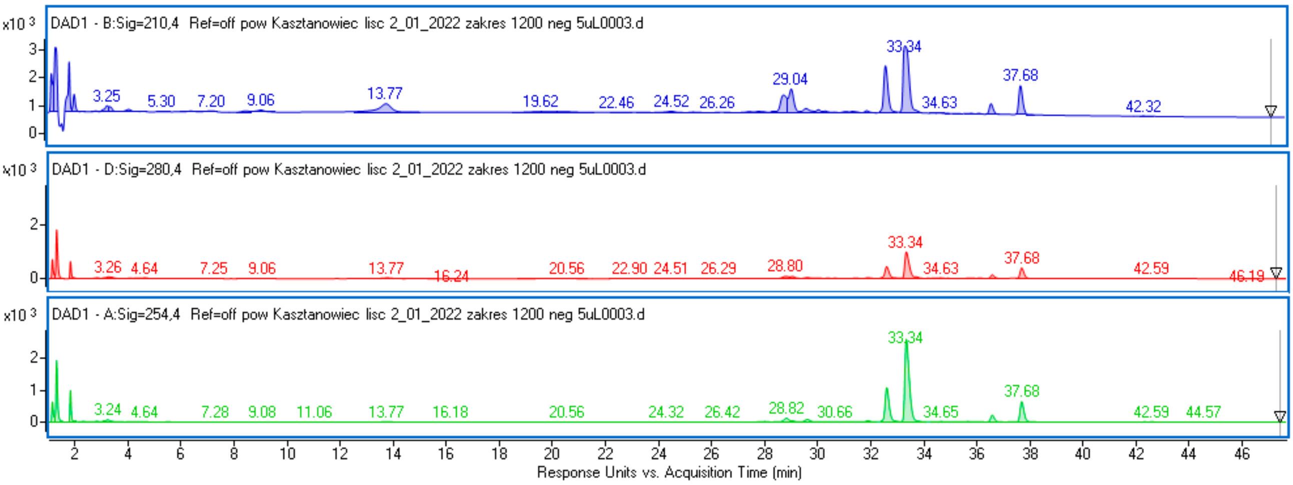

Figure S2. Representative chromatograms of *A. hippocastanum* control leaves recorded at 210 nm (blue), 280 (red) and 254 nm (green).

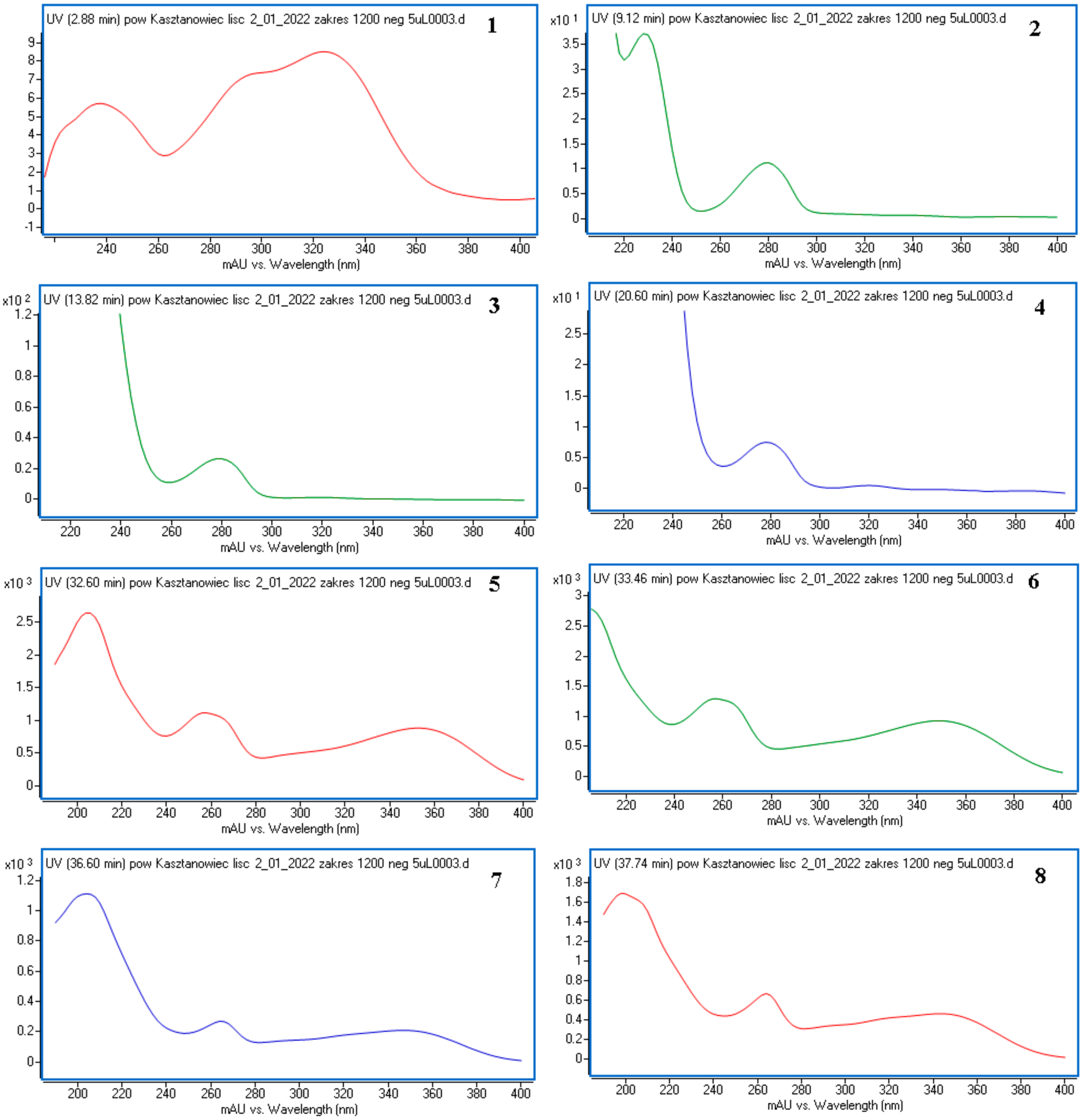

Figure S3. UV-Vis spectra of compounds identified in *A. hippocastanum* leaves. 1 - neochlorogenic acid, 2 - (−)-epicatechin, 3 - procyanidin trimer A-type, 4 - procyanidin tetramer A-type, 5 - quercetin-3-*O*-arabinoside, 6 - quercetin-3-*O*-rhamnoside, 7 - kaempferol-3-*O*-arabinoside, and 8 - kaempferol-3-*O*-rhamnoside.

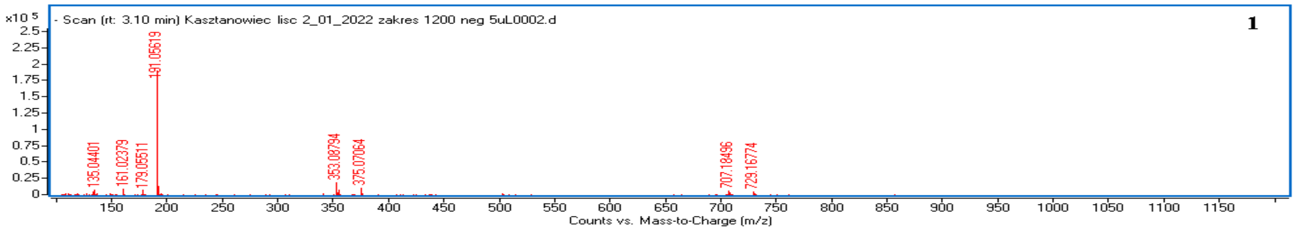
x10 5
- Scan (rt: 3.10 min) Kasztanowiec lisc 2_01_2022 zakres 1200 neg 5uL0002.d
1
135.04401
161.02379
179.05511
191.05619
353.08794
375.07064
707.18496
729.16774
Counts vs. Mass-to-Charge (m/z)

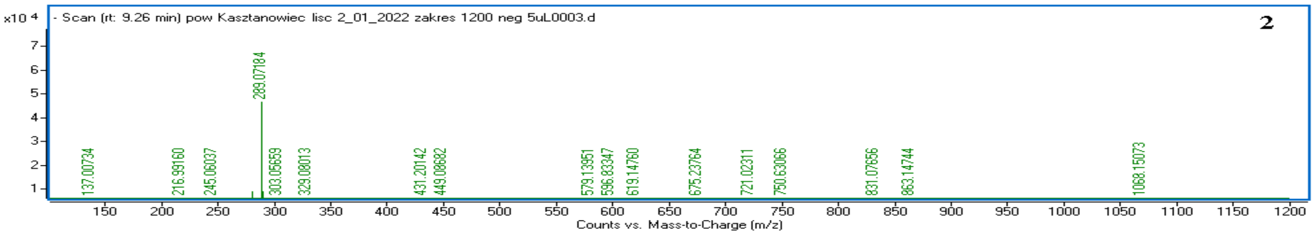
x10 4
- Scan (rt: 9.26 min) pow Kasztanowiec lisc 2_01_2022 zakres 1200 neg 5uL0003.d
2
137.00734
216.99160
245.06037
289.07184
303.05659
329.08013
431.20142
449.08682
579.13951
596.83347
619.14760
675.23764
721.02311
750.63066
831.07656
863.14744
1068.15073
Counts vs. Mass-to-Charge (m/z)

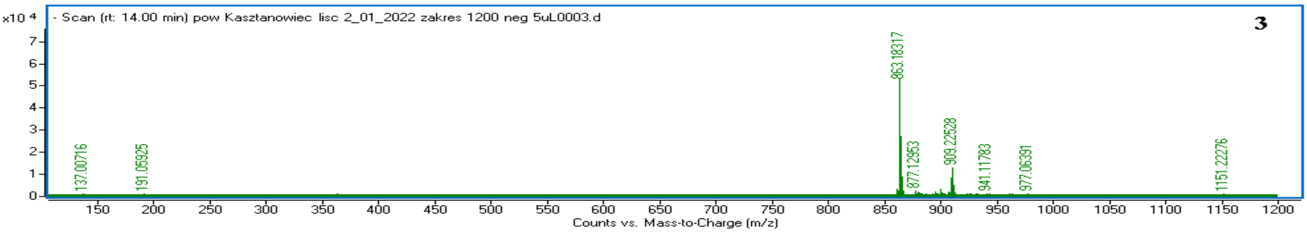
x10 4
- Scan (rt: 14.00 min) pow Kasztanowiec lisc 2_01_2022 zakres 1200 neg 5uL0003.d
3
137.00716
191.05925
863.18317
877.12953
909.22528
941.11783
977.06391
1151.22276
Counts vs. Mass-to-Charge (m/z)

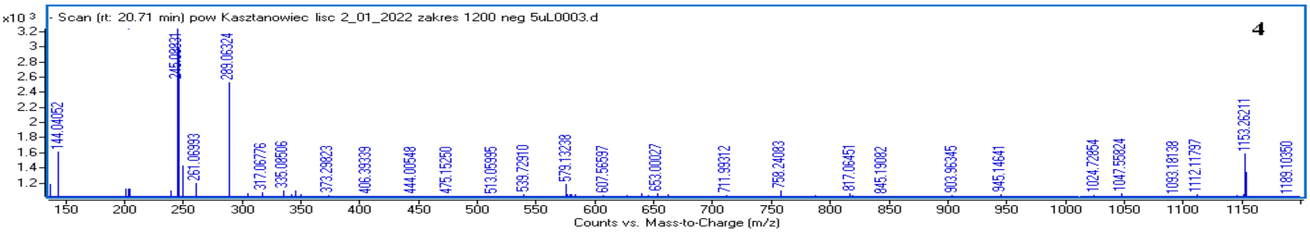
x10 3
- Scan (rt: 20.71 min) pow Kasztanowiec lisc 2_01_2022 zakres 1200 neg 5uL0003.d
4
144.04062
245.09831
261.06393
289.06324
317.06776
335.08506
373.29823
406.39339
444.00548
475.15250
513.05995
539.72910
579.13238
607.56597
653.00027
711.93312
758.24083
817.06451
845.19082
903.96345
945.14641
1024.72854
1047.55824
1093.18138
1112.11797
1153.26211
1189.10350
Counts vs. Mass-to-Charge (m/z)

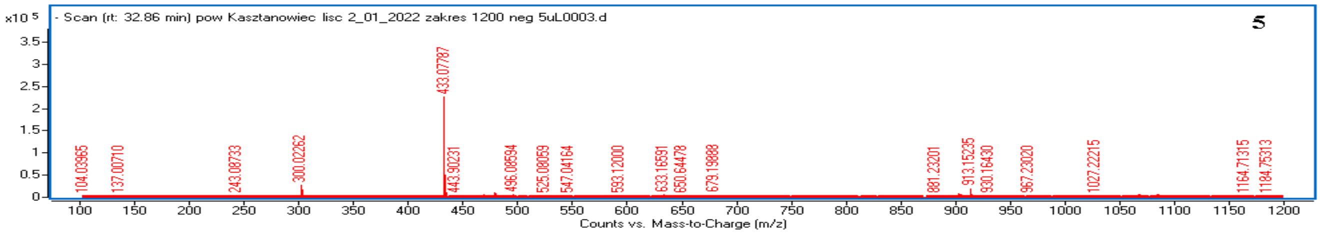
x10 5
- Scan (rt: 32.86 min) pow Kasztanowiec lisc 2_01_2022 zakres 1200 neg 5uL0003.d
5
104.03965
137.00710
243.08733
300.02262
433.07787
443.90231
496.08594
525.08059
547.04164
593.12000
633.16591
650.64478
679.19888
881.22201
913.15235
930.16430
967.23020
1027.22215
1164.71315
1184.75313
Counts vs. Mass-to-Charge (m/z)

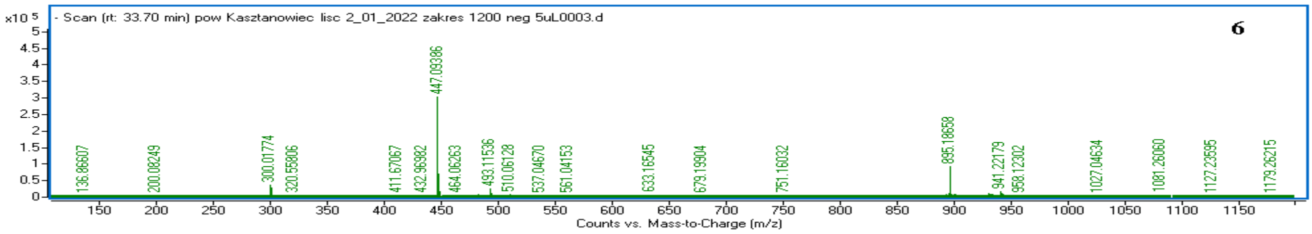
x10 5
- Scan (rt: 33.70 min) pow Kasztanowiec lisc 2_01_2022 zakres 1200 neg 5uL0003.d
6
136.86607
200.08249
300.01774
320.55806
411.67067
432.96382
447.09386
464.06263
493.11536
510.06128
537.04670
561.04153
633.16545
679.19904
751.16032
895.18658
941.22179
958.12302
1027.04634
1081.26060
1127.23595
1179.26215
Counts vs. Mass-to-Charge (m/z)

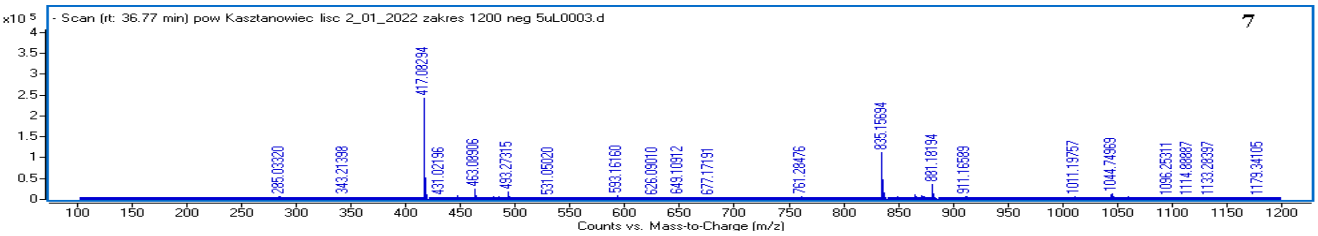
x10 5
- Scan (rt: 36.77 min) pow Kasztanowiec lisc 2_01_2022 zakres 1200 neg 5uL0003.d
7
285.03320
343.21398
417.08294
431.02196
463.08906
493.27315
531.05020
593.16160
626.09010
649.10912
677.17191
761.28476
835.15694
881.18194
911.16589
1011.19757
1044.74969
1096.25311
1114.88887
1133.28397
1179.34105
Counts vs. Mass-to-Charge (m/z)

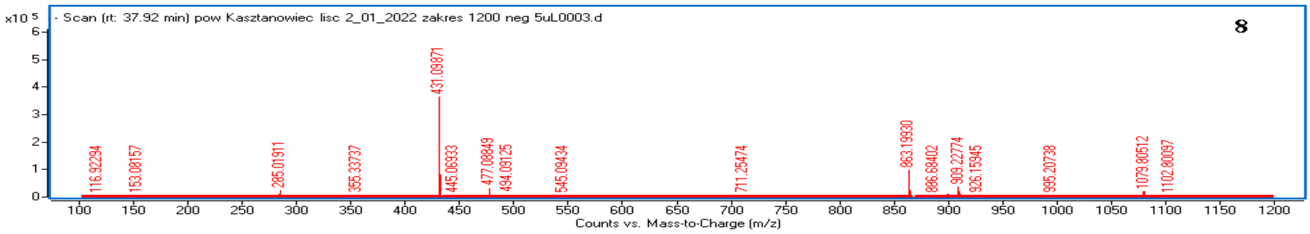
x10 5
- Scan (rt: 37.92 min) pow Kasztanowiec lisc 2_01_2022 zakres 1200 neg 5uL0003.d
8
116.92294
153.08157
285.01911
355.33737
431.09871
445.06933
477.08849
494.09125
545.09434
711.25474
863.19930
886.68402
909.22774
926.15945
995.20738
1079.80512
1102.80097
Counts vs. Mass-to-Charge (m/z)

Figure S4. MS spectra of compounds identified in *A. hippocastanum* leaves (in the negative ionization mode). 1 - neochlorogenic acid, 2 - (−)-epicatechin, 3 - procyanidin trimer A-type, 4 - procyanidin tetramer A-type, 5 - quercetin-3-*O*-arabinoside, 6 - quercetin-3-*O*-rhamnoside, 7 - kaempferol-3-*O*-arabinoside, and 8 - kaempferol-3-*O*-rhamnoside.

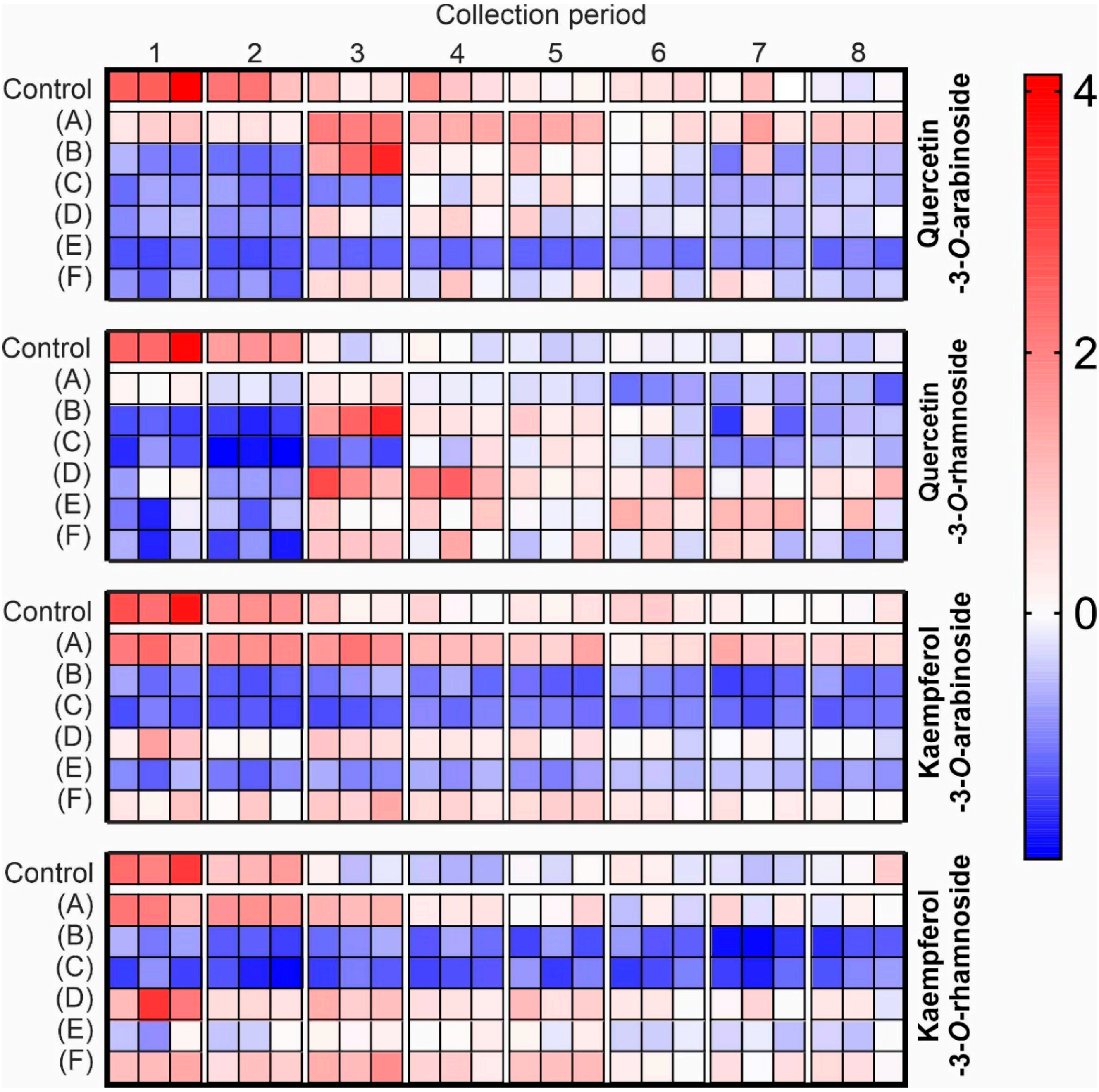

Figure S5. Heat map of quercetin and kaempferol derivative contents in the leaves of *A. hippocastanum* trees (control and symptomatic A-F) measured at the seventh collection period. The higher the level of the measured compound, the more intense the red color. The lower the level of the compound, the more intense the blue color.

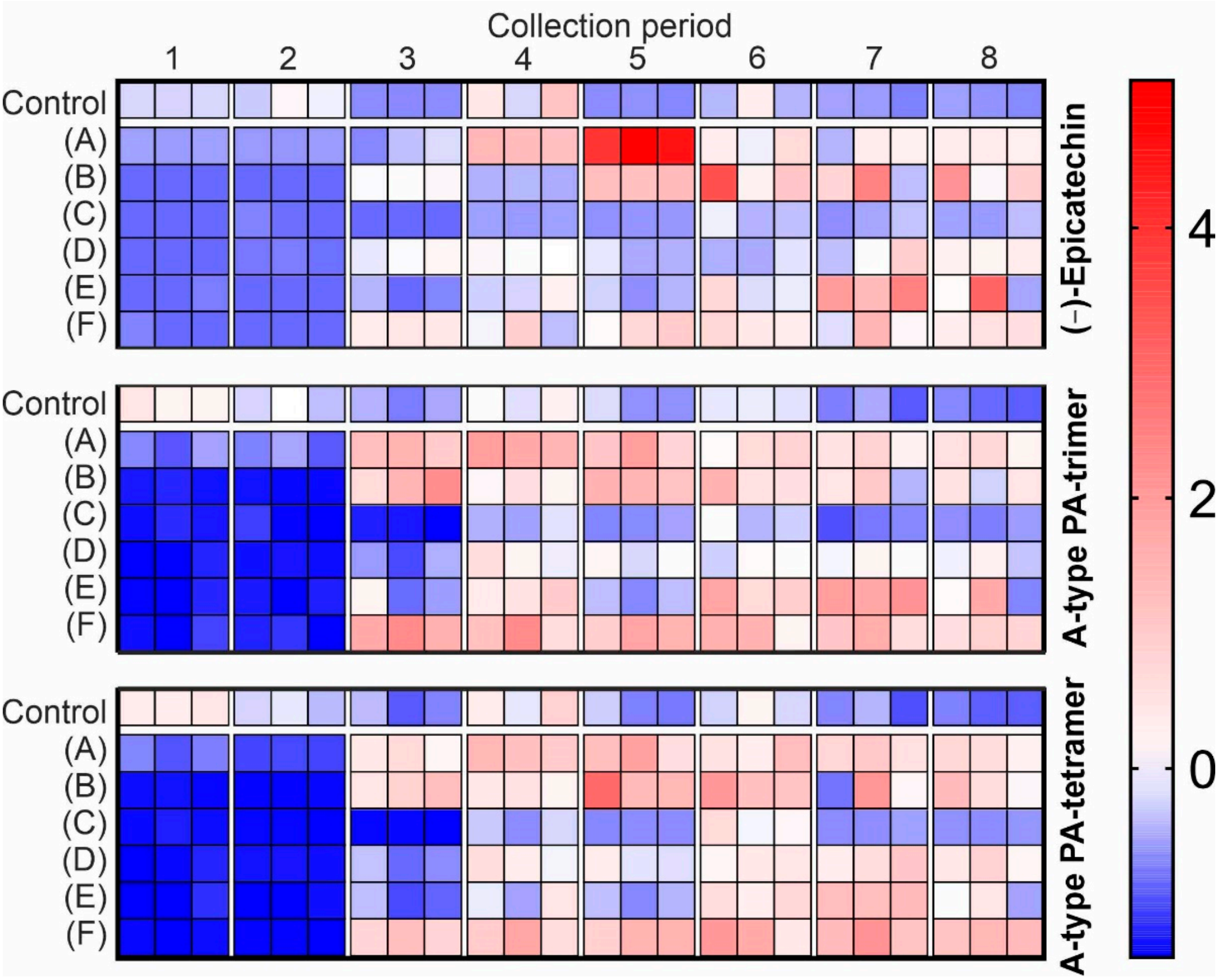

Figure S6. Heat map of epicatechin and procyanidin contents in the leaves of *A. hippocastanum* trees (control and symptomatic A-F) measured at the seventh collection period. The higher the level of measured compound, the more intense the red color. The lower the level of the compound, the more intense the blue color.

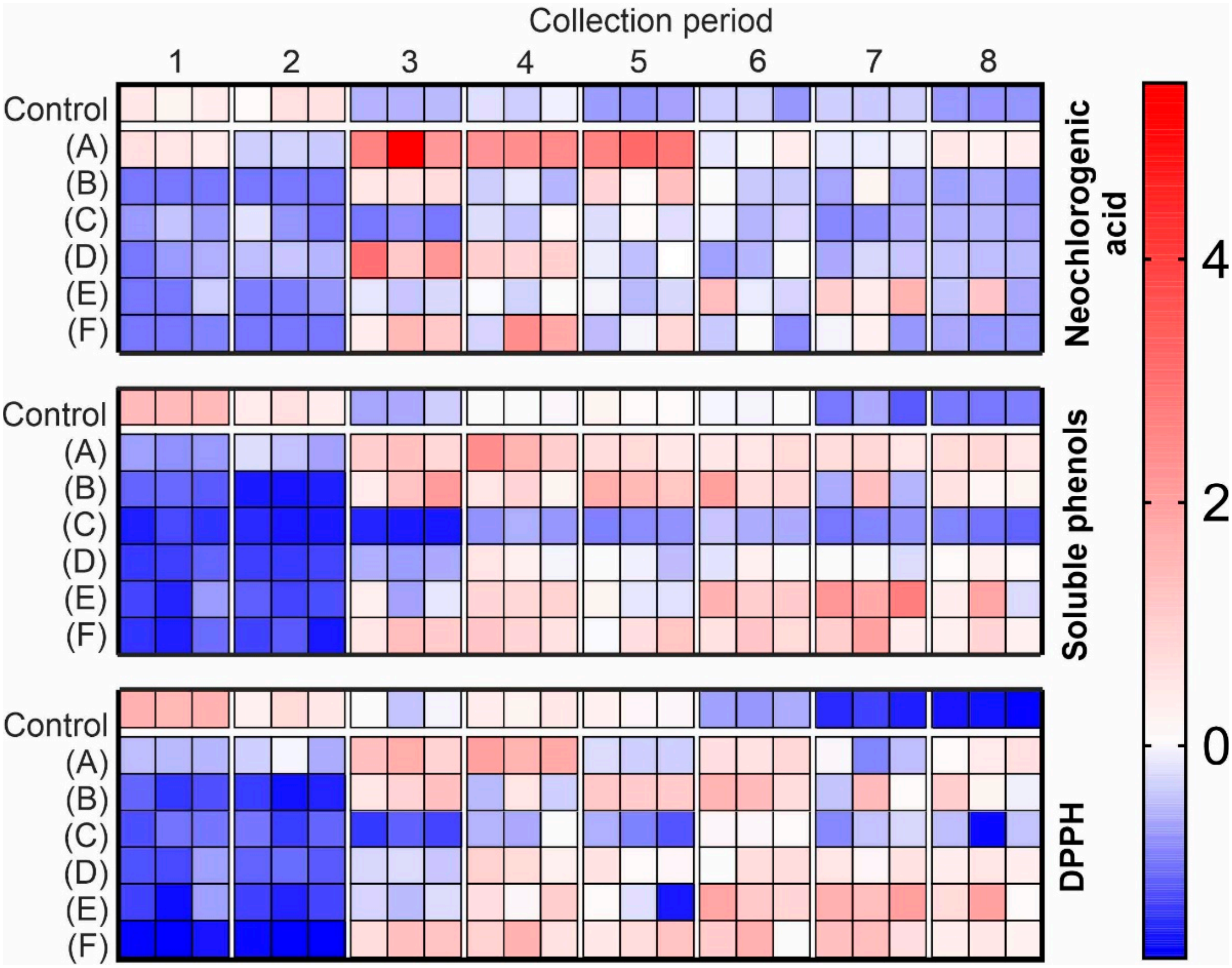

Figure S7. Heat map of the contents of neochlorogenic acid and soluble phenols and the DPPH level in the leaves of *A. hippocastanum* trees (control and symptomatic A-F) measured at the seventh collection period. The higher the level of measured compound, the more intense the red color. The lower the level of the compound, the more intense the blue color.

Table S1. Selected meteorological parameters in Lublin: mean temperature and precipitation from April to August [37].

Parameters	April	May	June	July	August
Mean T [ºC]	9.4	12.8	21.3	18.3	19.7
Mean Max T [ºC]	14.8	17.7	27.3	24.5	26.0
Mean Min T [ºC]	4.2	8.8	15.2	12.9	14.1
Precipitation days					
≥ 0.1 mm	8	16	4	11	9
≥ 1 mm	6	11	2	6	8
≥ 5 mm	2	7	1	2	6
≥ 10 mm	1	2	1	0	2
≥ 20 mm	0	2	0	0	1

Table S2. Formulas of the calibration curves for neochlorogenic acid, (−)-epicatechin and the corresponding flavonoid aglycones (quercetin and kaempferol).

Compound	Formula
neochlorogenic acid	y = 47789109.3 x + 3028.5; R^2 = 0.9998
epicatechin	y = 244926385.4 x - 11887.0; R^2 = 0.9999
quercetin	y = 40258231.2 x - 975653.5; R^2 = 0.9979
kaempferol	y = 45051376.1 x - 923586.9; R^2 = 0.9922
